# Supplementary figures and images for: Role of endoplasmic reticulum stress in impaired neonatal lung growth and bronchopulmonary dysplasia
Source: PLoS One. 2022 Aug 26;17(8):e0269564. doi: 10.1371/journal.pone.0269564 (PMC9417039; doi:10.1371/journal.pone.0269564)

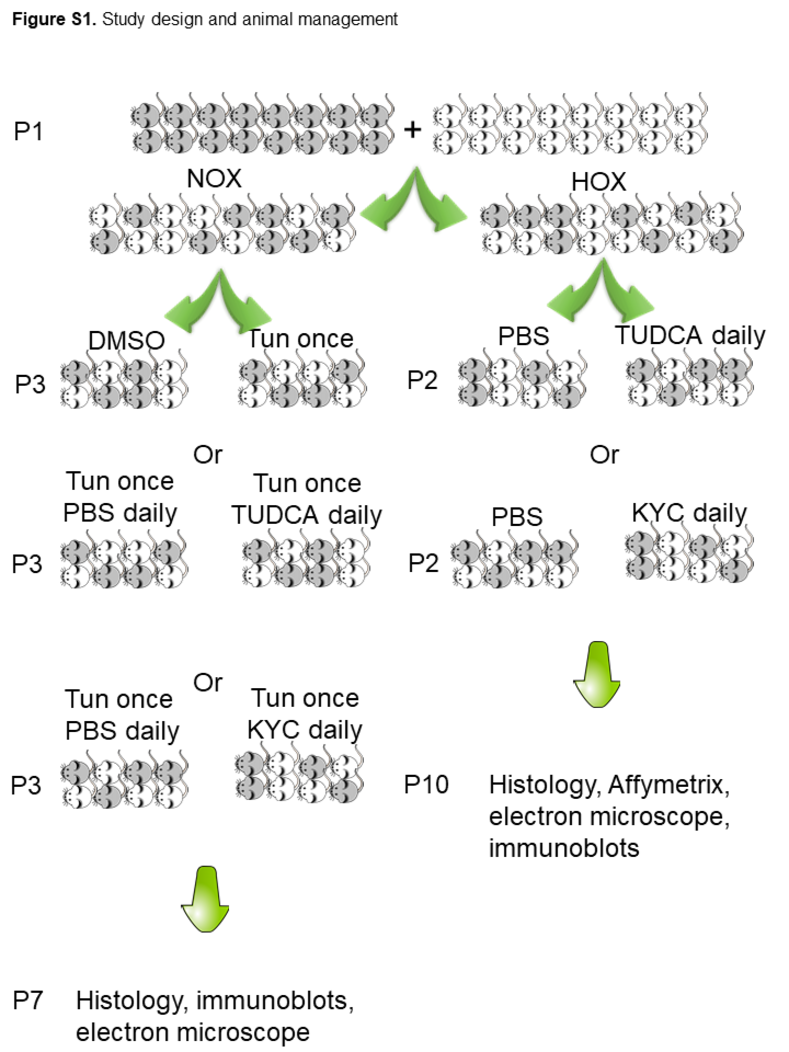

Supplement: S1 Fig — Rat pups from 2–4 dams were mixed and then randomly allocated into different treatment groups at P1. After randomization, the pups were raised in either NOX or HOX with nursing dams. Tunicamycin 0.01 mg/kg was given i.p. once at P3. Tauroursodeoxycholic acid 100 mg/kg/dm i.p., or KYC 10 mg/kg/d i.p., was given once daily starting at P2. Lungs were obtained at P7 for tunicamycin studies but at P10 for HOX studies. (TIF) [file pone.0269564.s001.tif]

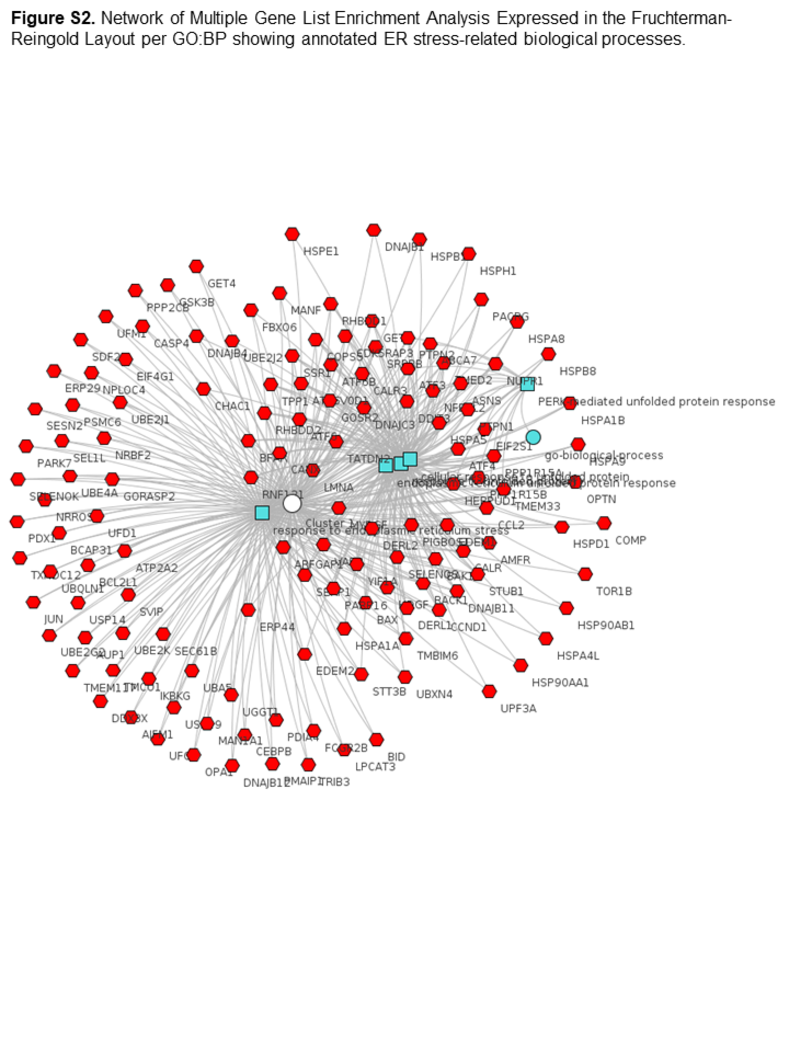

Supplement: S2 Fig — Multiple gene enrichment analysis shows multiple ER stress-related biological processes are annotated by ToppCluster according to the Gene Ontology Biological Processes (GO-BP). (TIF) [file pone.0269564.s002.tif]

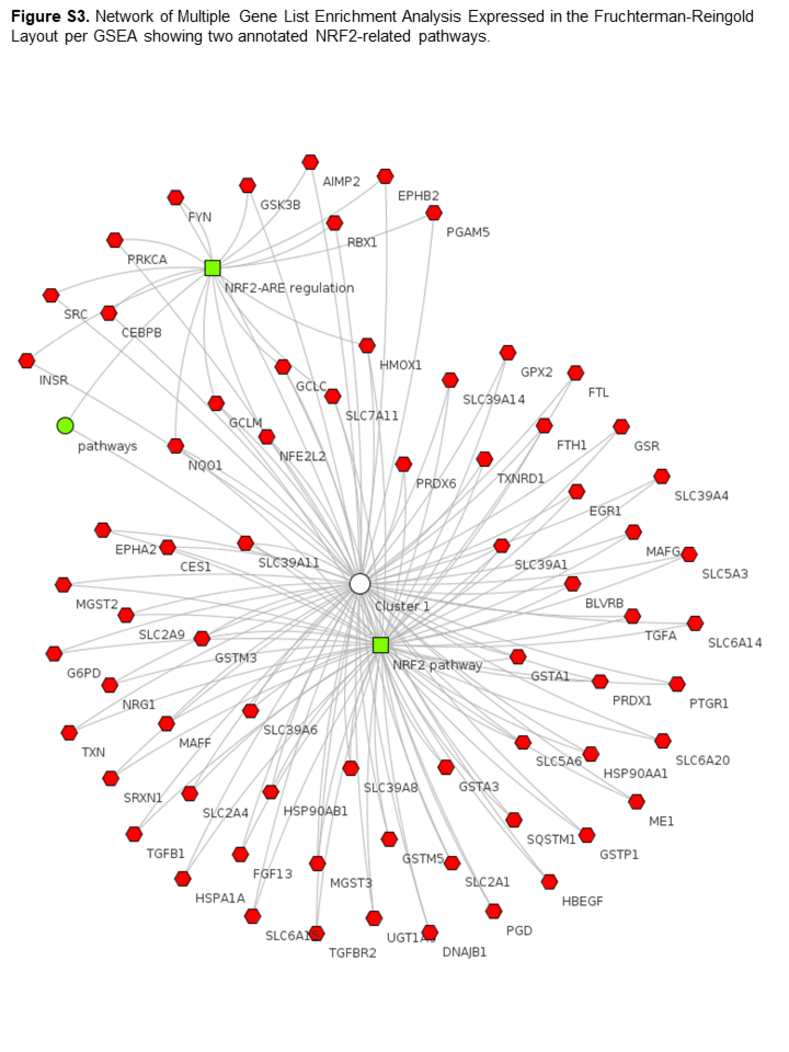

Supplement: S3 Fig — Multiple gene enrichment analysis shows ToppCluster annotates two NRF2-related pathway processes according to the WikiPathways. (TIF) [file pone.0269564.s003.tif]

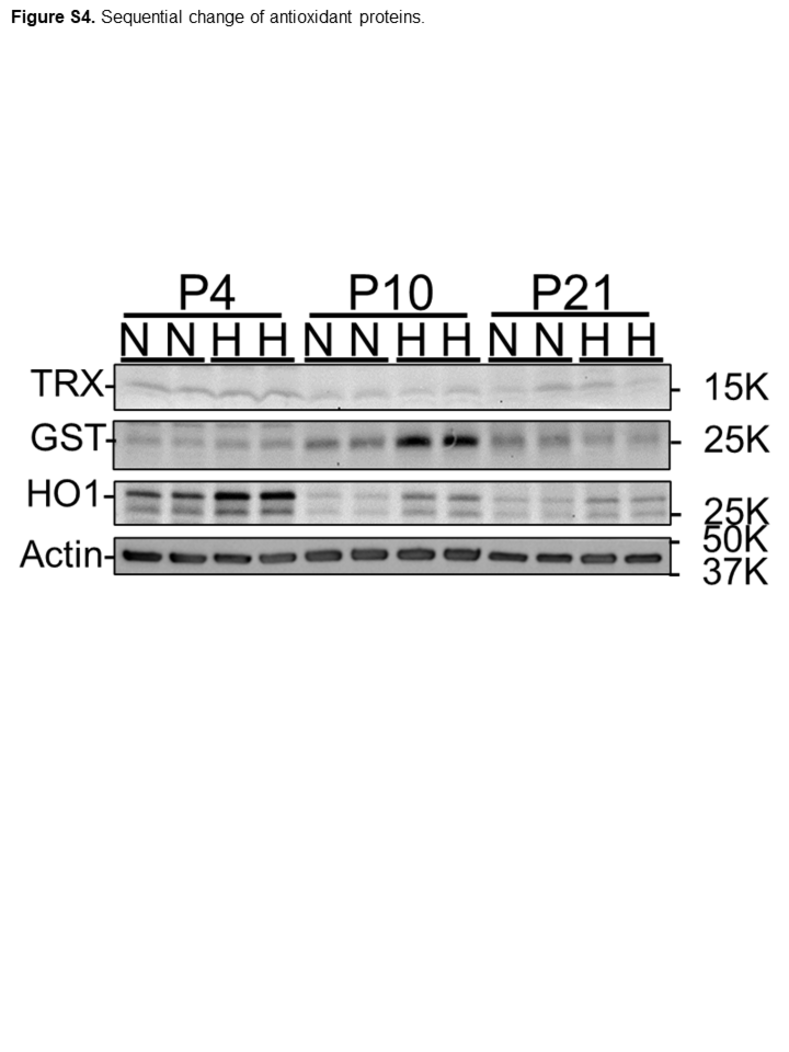

Supplement: S4 Fig — Expressions of NRF2 downstream antioxidants (thioredoxin-1, glutathione-S-transferase, and heme oxygenase-1) are increased in HOX neonatal rat lungs as early as P4; some of them (GST1 and HO1) persist until P10, or (HO1) even persist after recovery in room air for 11 days at P21. (TIF) [file pone.0269564.s004.tif]
