# Supplementary material for: Role of endoplasmic reticulum stress in impaired neonatal lung growth and bronchopulmonary dysplasia
Source: PLoS One. 2022 Aug 26;17(8):e0269564. doi: 10.1371/journal.pone.0269564 (PMC9417039; doi:10.1371/journal.pone.0269564)
Supplement: S1 Table — Detailed information was not available for this age- and sex-matched deidentified specimens. There were 10 cases, 6 males and 4 females, for each group. Half (5 out of 10) of the matched controls died of inoperable complex congenital heart diseases, and none received extensive mechanical ventilator or supplemental oxygen support. (PDF) [file pone.0269564.s005.pdf]

Table S1. Age, sex, and diagnosis of the autopsy specimens.

BPD			Control		
**Age**	**Sex**	**Diagnosis**	**Age**	**Sex**	**Diagnosis**
5W	M	BPD	1M	M	Wolf-Hirschhorn and renal dysgenesis
11W	F	BPD	5M	F	HLHS
10W	M	BPD	10W	M	HLHS
3M	M	BPD	3M	M	Congenital heart disease
8M	F	BPD	7M	F	Liver failure/PFIC
4M	F	BPD	4M	F	Congenital heart disease
5W	M	BPD	25D	M	Hypoxic ischemic encephalopathy
3M	M	BPD	3M	M	Osteogenesis imperfecta
7W	F	BPD	2M	F	Congenital heart disease
16M	M	BPD	13M	M	Hemophagocytic lymphohistiocytosis

HLHS: hypoplastic left heart syndrome; PFIC: progressive familial intrahepatic cholestasis (Byler disease).
