# Supplementary material for: Role of endoplasmic reticulum stress in impaired neonatal lung growth and bronchopulmonary dysplasia
Source: PLoS One. 2022 Aug 26;17(8):e0269564. doi: 10.1371/journal.pone.0269564 (PMC9417039; doi:10.1371/journal.pone.0269564)
Supplement: S2 Table — (PDF) [file pone.0269564.s006.pdf]

Table S2. Transcriptomes enriched in two NRF2-related pathways as annotated by the ToppCluster using WikiPathways as the data source.

WikiPathway	Systemic Name	p-value	Transcriptomes identified
M39761	NRF2-ARE regulation	$2.82x10^{-6}$	AIMP2 CEBPB EPHB2 FYN GCLC GCLM GSK3B HMOX1 INSR NFE2L2 NQO1 PGAM5 PRKCA RBX1 SLC7A11 SRC
M39454	NRF2 pathway	$3.60x10^{-6}$	BLVRB CES1 DNAJB1 EGR1 EPHA2 FGF13 FTH1 FTL G6PD GCLC GCLM GPX2 GSR GSTA1 GSTA3 GSTM3 GSTM5 GSTP1 HBEGF HMOX1 HSP90AA1 HSP90AB1 HSPA1A MAFF MAFG ME1 MGST2 MGST3 NFE2L2 NQO1 NRG1 PGD PRDX1 PRDX6 PTGR1 SLC2A1 SLC2A4 SLC2A9 SLC39A1 SLC39A11 SLC39A14 SLC39A4 SLC39A6 SLC39A8 SLC5A3 SLC5A6 SLC6A14 SLC6A15 SLC6A20 SLC7A11 SQSTM1 SRXN1 TGFA TGFB1 TGFBR2 TXN TXNRD1 UGT1A6
