# Supplementary material for: Role of endoplasmic reticulum stress in impaired neonatal lung growth and bronchopulmonary dysplasia
Source: PLoS One. 2022 Aug 26;17(8):e0269564. doi: 10.1371/journal.pone.0269564 (PMC9417039; doi:10.1371/journal.pone.0269564)

raw_Figure 2A

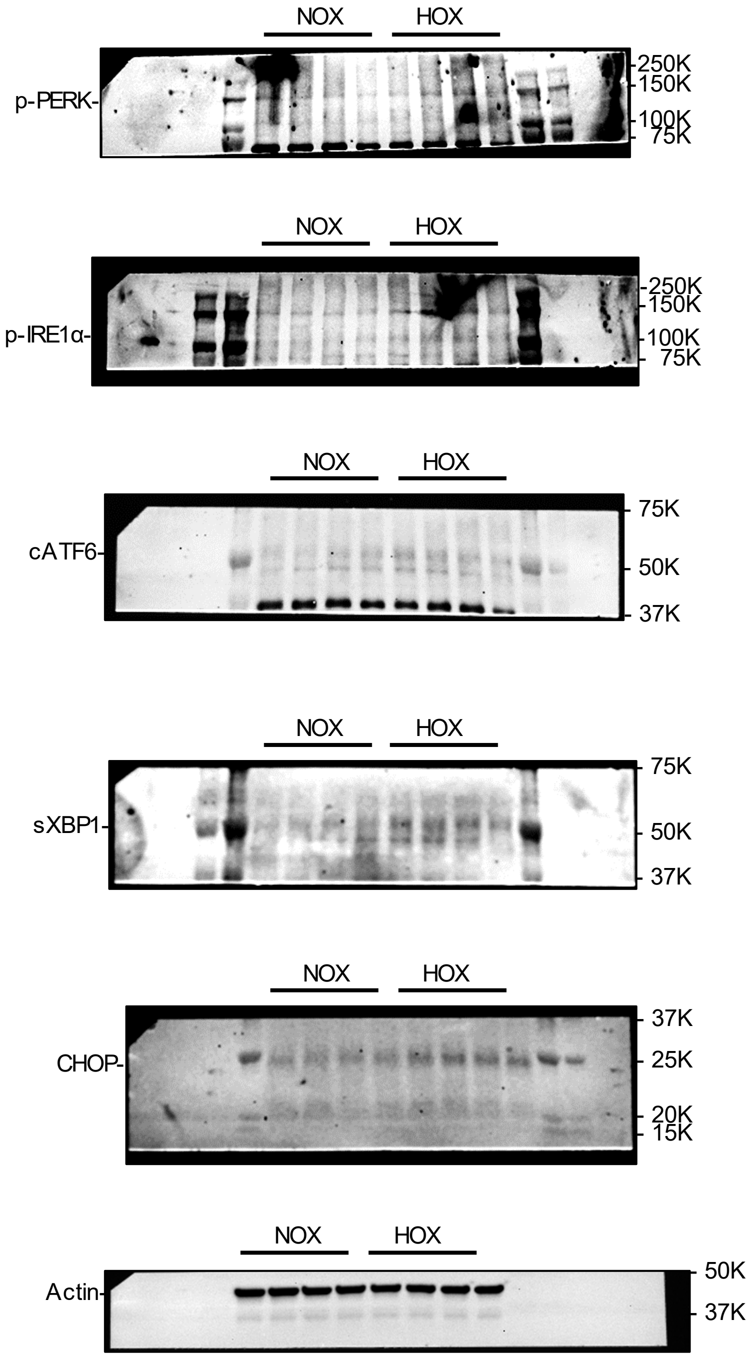

raw_Figure 3B

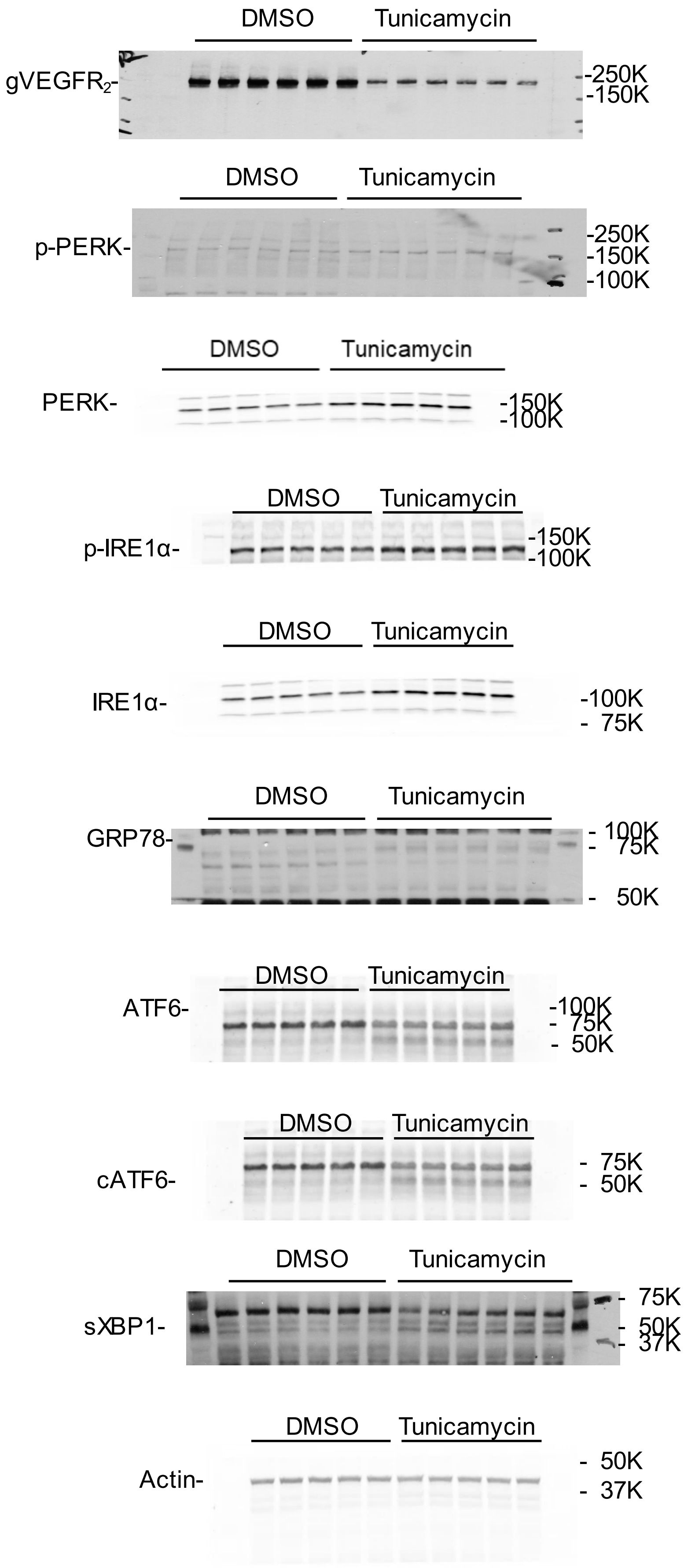

raw_Figure 3C

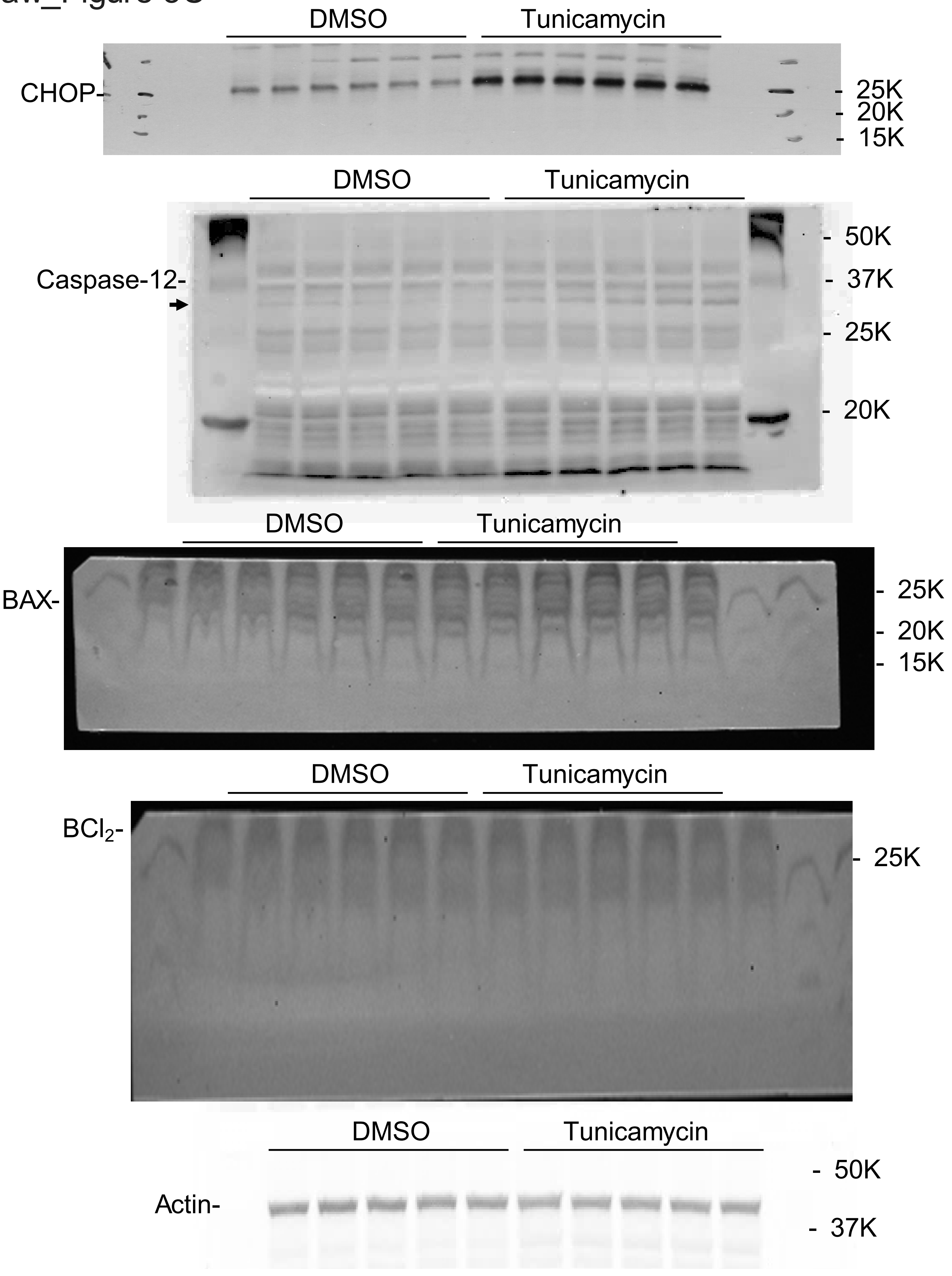

raw_Figure 4A

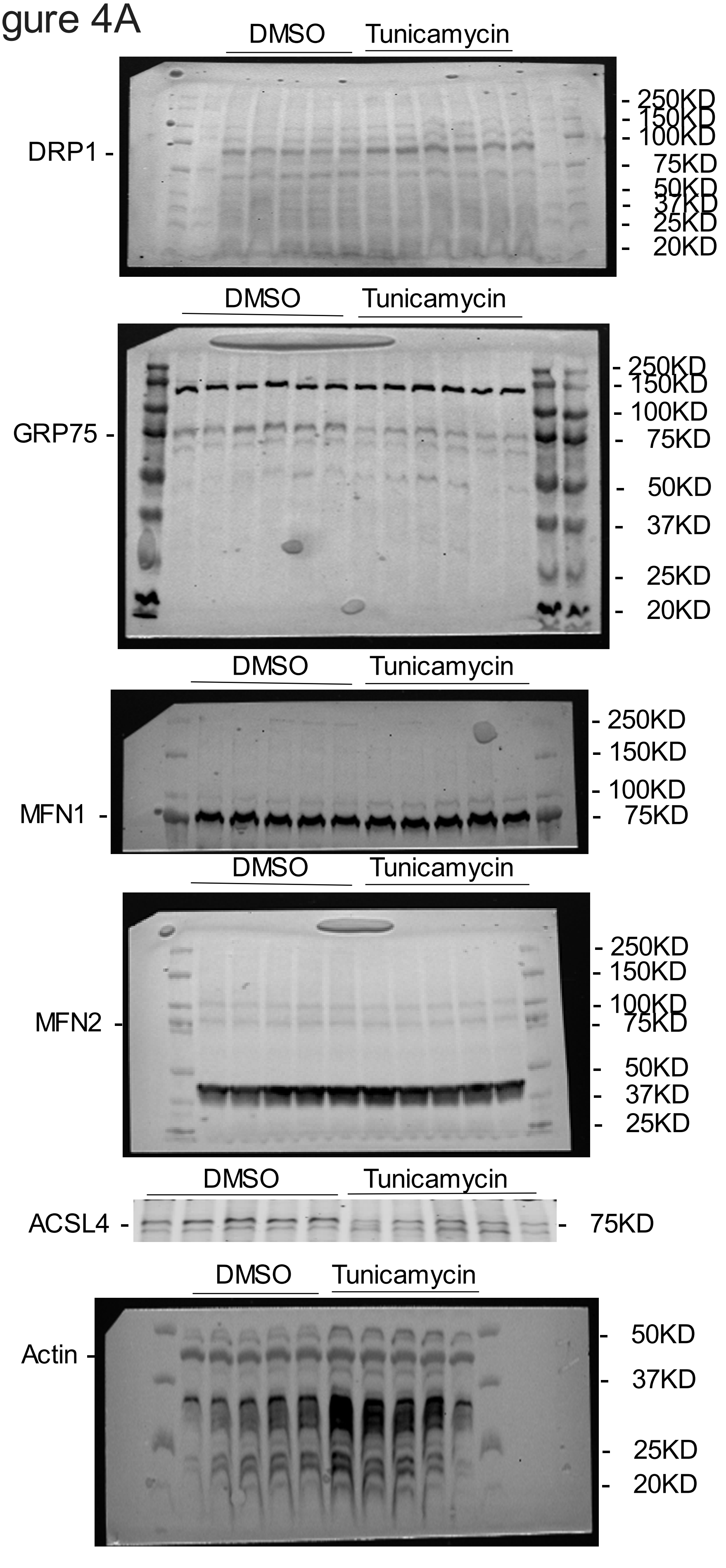

raw_Figure 4D

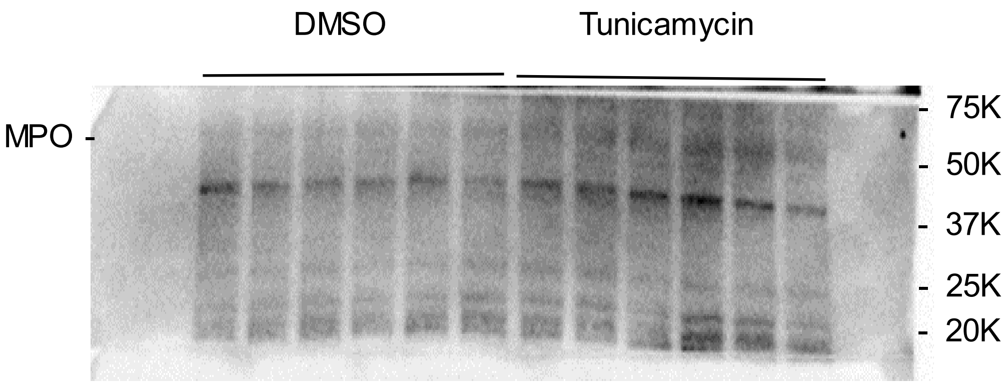

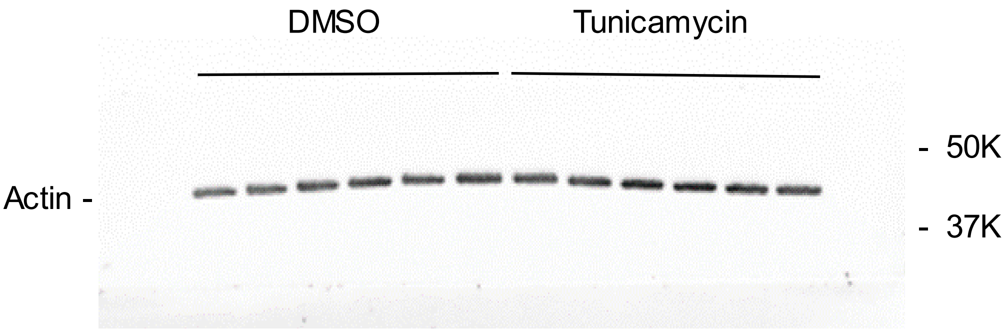

raw_Figure 6A

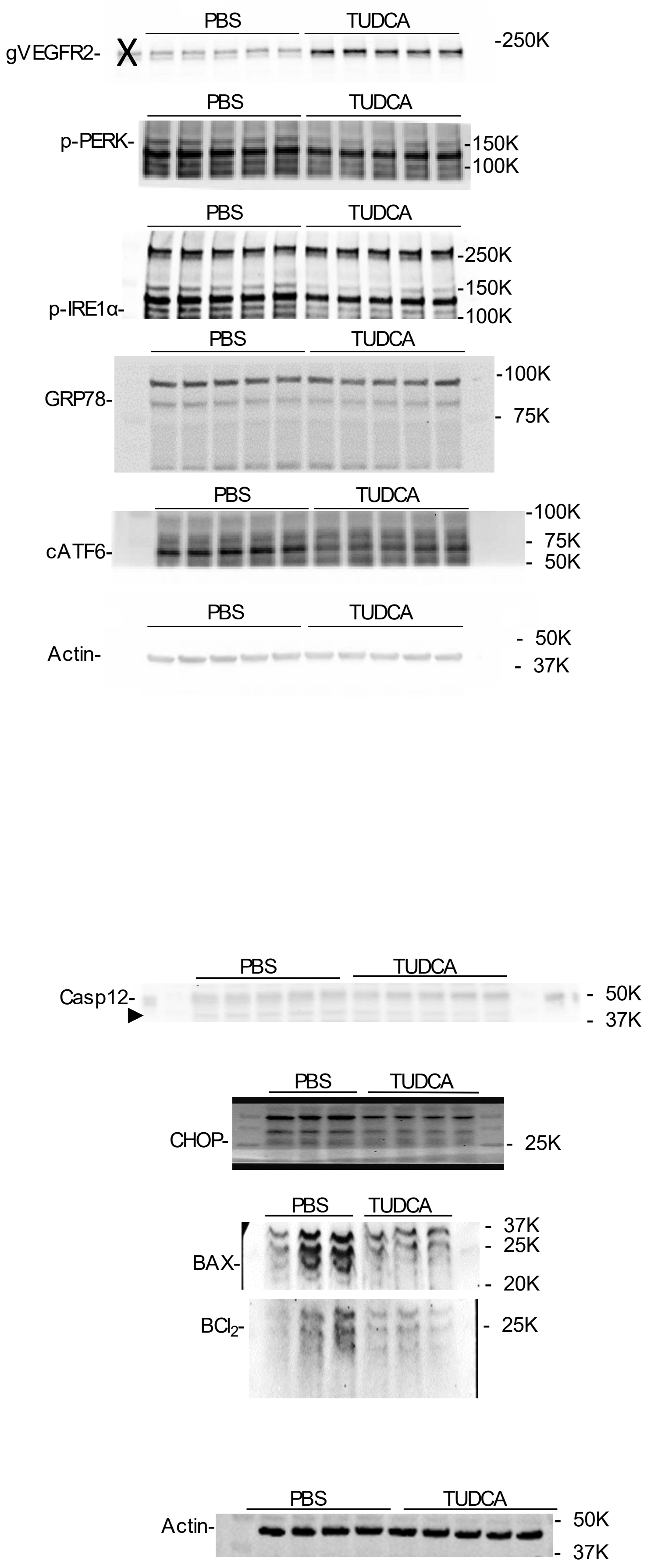

raw_Figure 7A

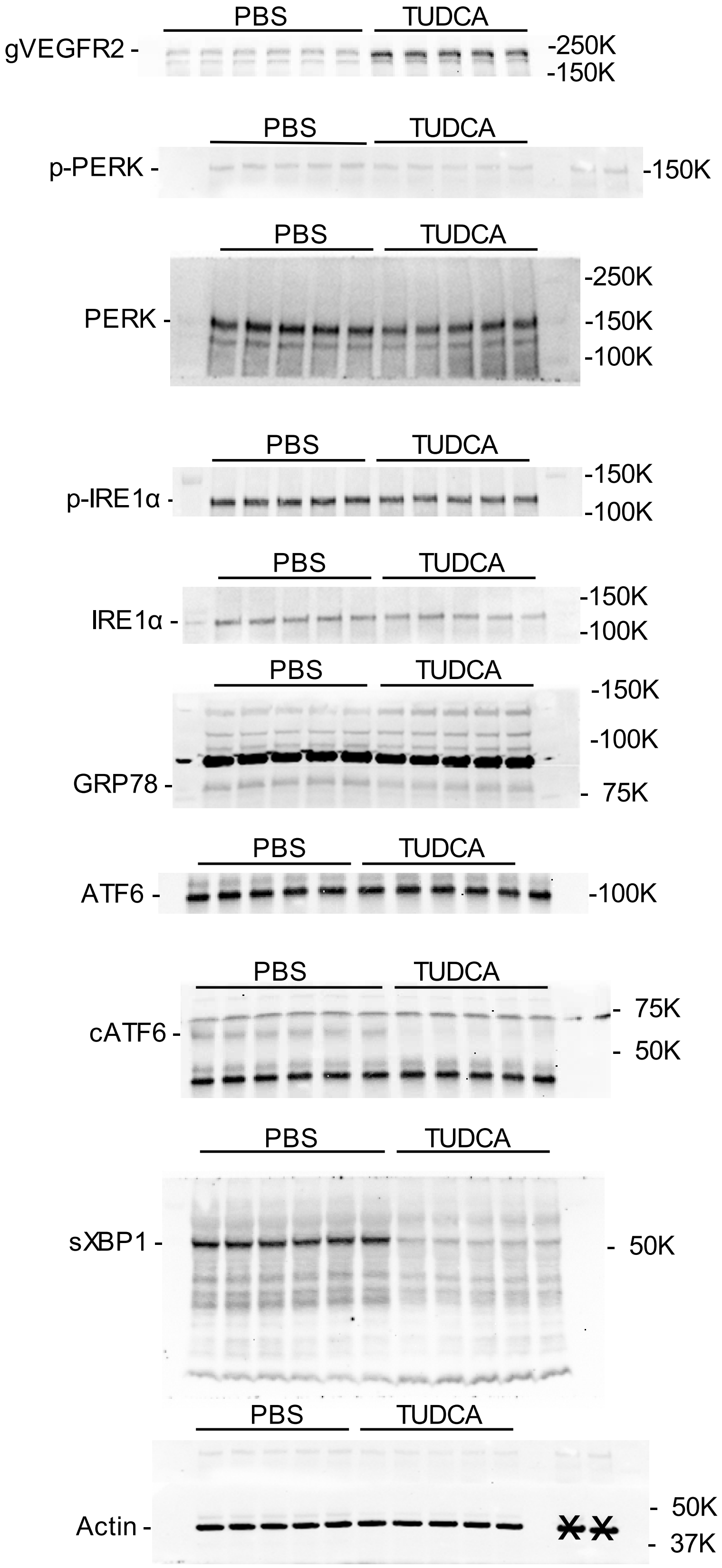

raw_Figure 7B

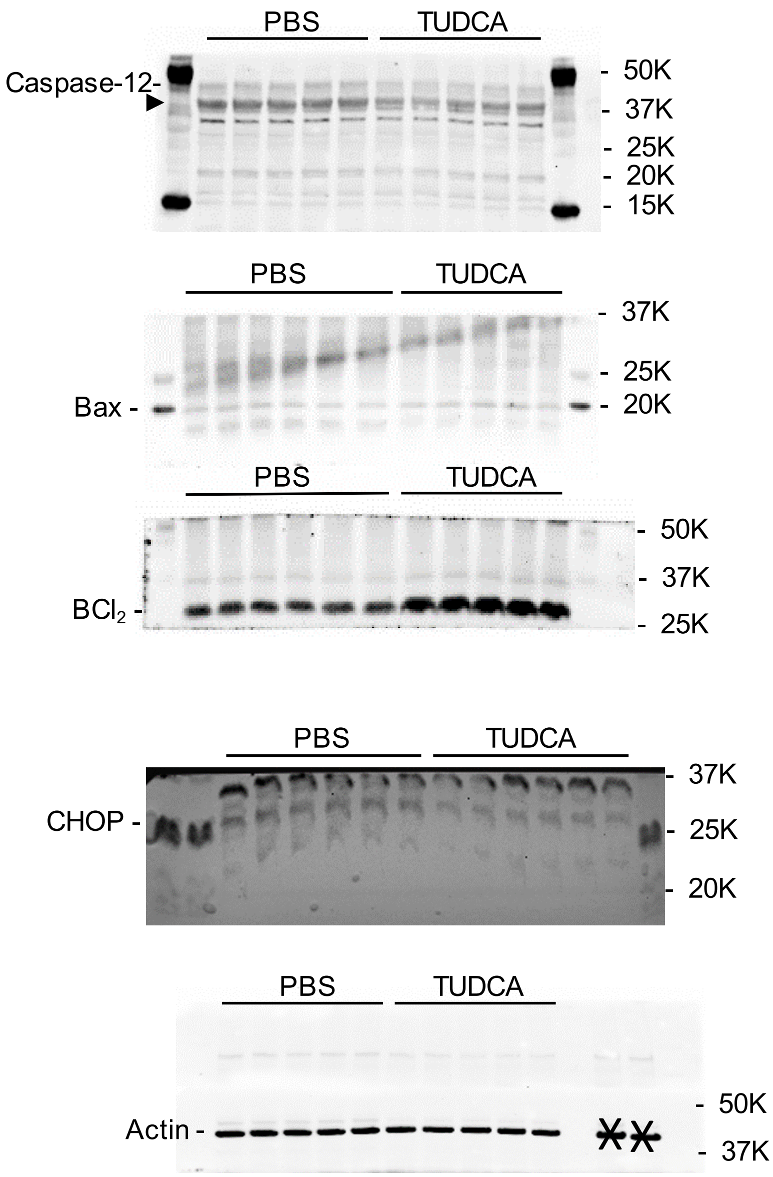

raw_Figure 8A

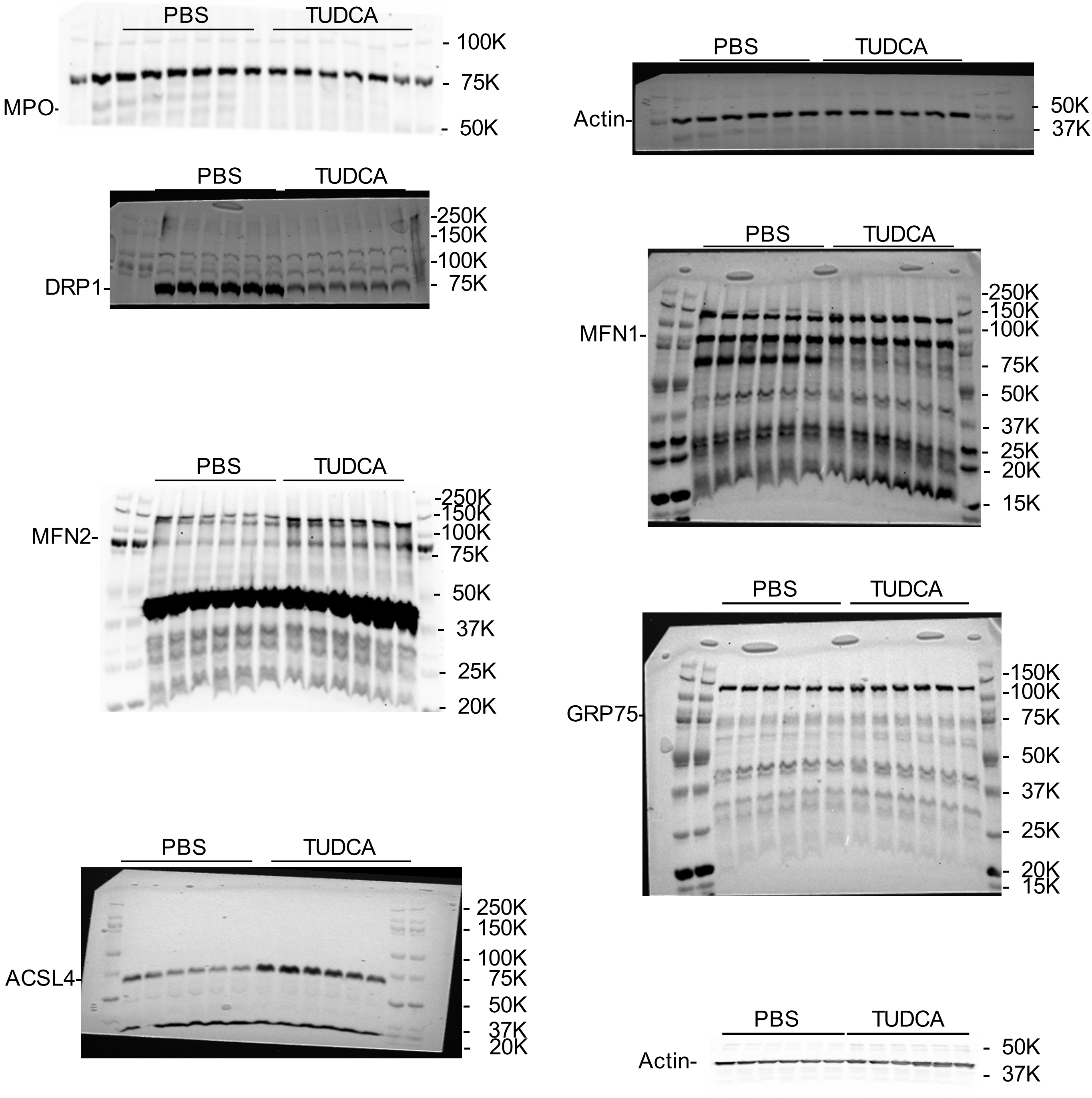

raw_Figure 10A

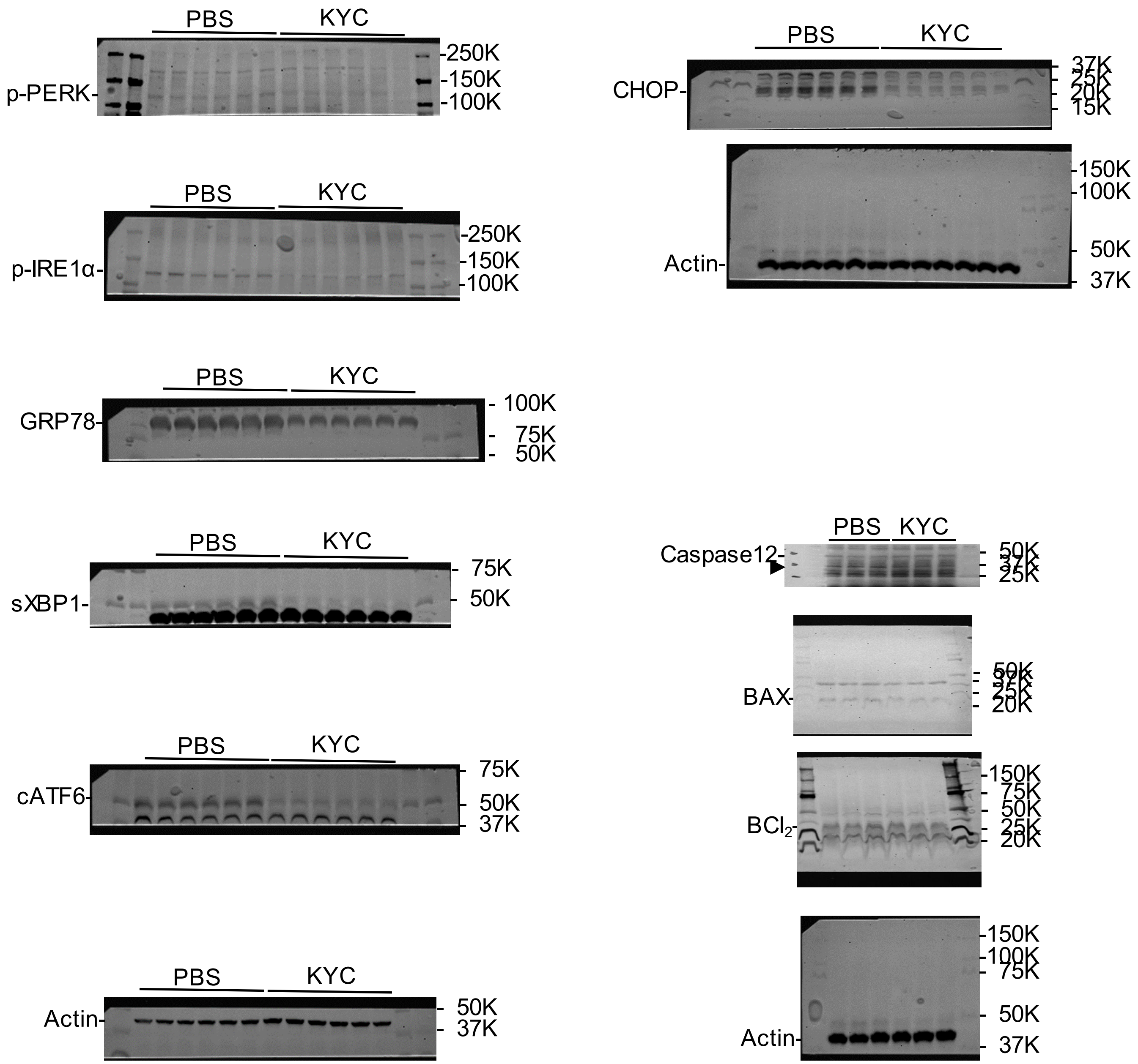

raw_Figure 10C

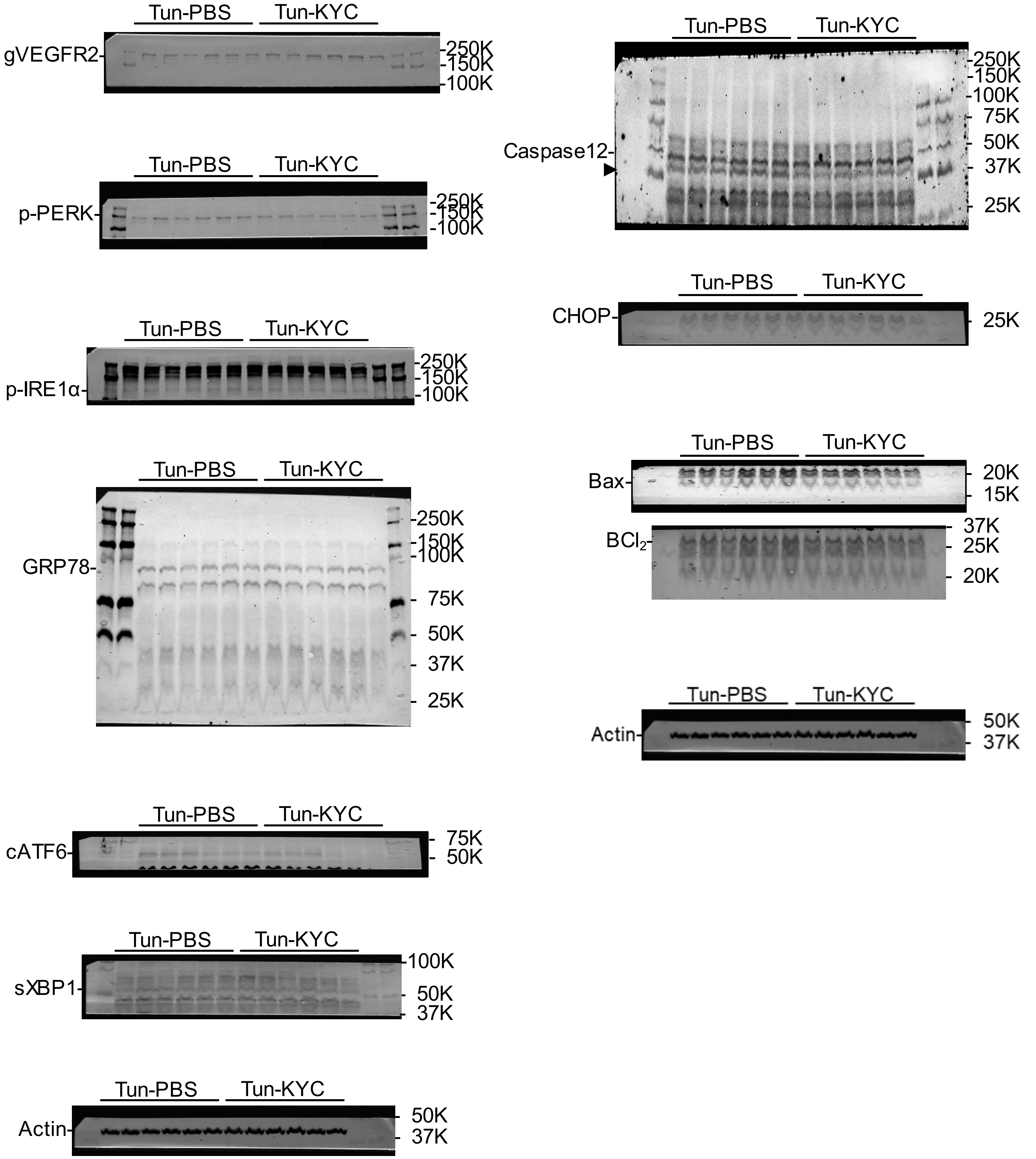

raw_Figure 10D

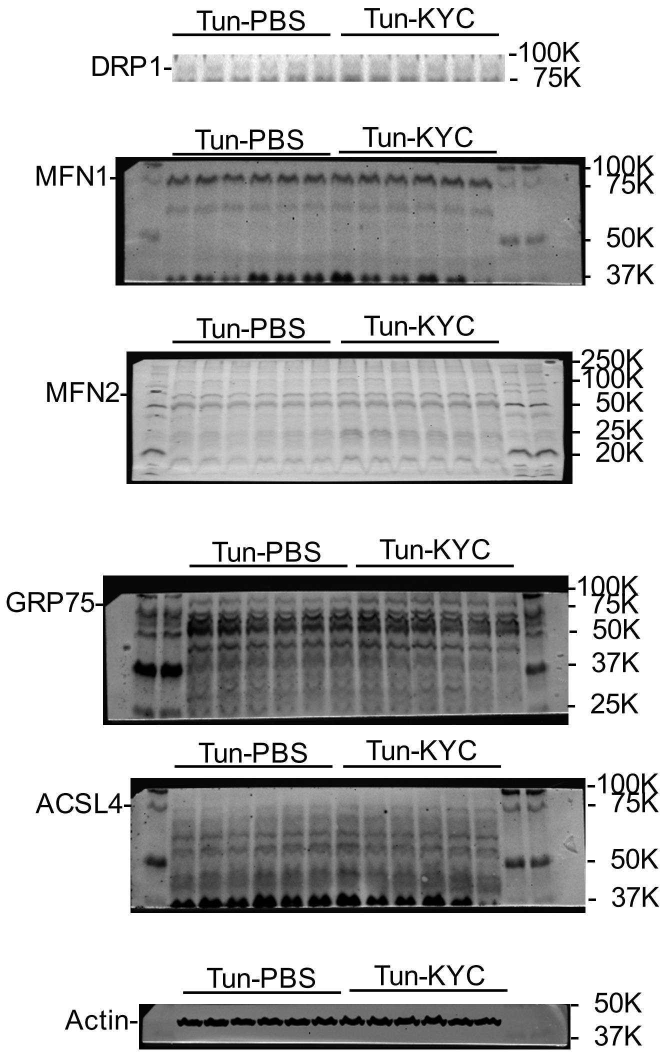

raw_Figure 10E

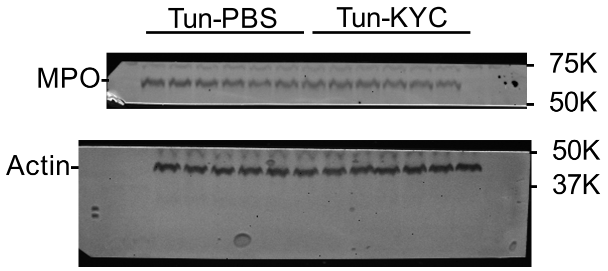

Supplement: S1 Raw images — (PDF) [file pone.0269564.s007.pdf]
