# Supplementary material for: Role of endoplasmic reticulum stress in impaired neonatal lung growth and bronchopulmonary dysplasia
Source: PLoS One. 2022 Aug 26;17(8):e0269564. doi: 10.1371/journal.pone.0269564 (PMC9417039; doi:10.1371/journal.pone.0269564)
Supplement: Attachment — Submitted filename: Review Article PLOS ONE AD.docx [file pone.0269564.s008.docx]

Dear Professor Dr. Bader,

I would like to thank you for the opportunity to review the submitted manuscript titled: “Role of endoplasmic reticulum stress in impaired neonatal lung growth and bronchopulmonary dysplasia”.

I found this article interesting and complicated to read. I can summarize the manuscript as follows:

Extreme premature neonates due to their immaturity often require mechanical ventilatory support and hyperoxic exposure to maintain life. They can develop as a complication to these exposures, Bronchopulmonary Dysplasia, which is a grave condition characterized phenotypically by alveolar simplification and vascular rarefaction. Excessive oxidative stress with inadequate anti-oxidant defenses and immature inflammatory responses are believed to be the main pathophysiological drivers to the establishment of the condition. The authors of this manuscript attempt to provide -without a clear hypothesis - a connection between oxidative and inflammatory pathways via endoplasmic reticulum stress to the development of BPD with the employment of both a human and an animal model.

In their experimental design, they first assess ESR stress in human lung autopsy specimens of children with the diagnosis of BPD vs controls.

They proceed with their animal experimental design with the independent application -in parallel - of two stressors. One is with hyperoxic exposure and the other is via a known pharmacological inducer of ERS which is tunicamycin. With their experiments the authors are able to show that both stressors induce ERS and apoptosis leading to lung phenotypes that include decreased alveolarization, vascularization and increased inflammatory cell infiltration. After the stressor application, they followed their experiments by providing pharmacological agents that serve as potential therapeutics that target the endoplasmic reticulum stress, providing information to prove that these agents can rescue from the pathologic phenotype.

In detail, newborn rats were exposed to hyperoxia in an established experimental model of Bronchopulmonary Dysplasia. The authors were able to elucidate lung morphologies phenotypically similar in appearance with histopathological findings of bronchopulmonary dysplasia, with diminished alveolarization, decreased vascularization and increased inflammatory cell infiltration. Survival was also found decreased. With the employment of various techniques, they provide ample data to suggest that Hyperoxic exposure does lead to ERS which is attenuated via TUDCA (Tauroursodeoxycholic acid, an ER chaperone that can alleviate ERS) and KYC (N-acetyl83 lysyltyrosylcysteine amide, a proposed anti-inflammatory agent) administration.

The authors were able to show similar results with their parallel animal model using tunicamycin as the stressor. Tunicamycin decreased survival, weight gain and induced a lung phenotype similar to the previous model. The effects seem to be mitigated through TUDCA and KYC administration as well. Even though the experiments are in parallel and reveal similar outcomes it led the authors to conclude that the ERS response is fundamental for the development of BPD.

This conclusion is by association as no concomitant exposure to both stressors was utilized nor the exact techniques were employed between the parallel models. The authors rely heavily on their previous work which these experiments supplement. The novelty of this paper is the use of these potential therapeutic agents in the same experimental design, however they are reported and compared individually. Additional critical details on the methods need to be communicated.

Authors display the following information:

Survival: Yes

Population: Partially

Weight: Yes

Sex: No

Experimental Design Graph: No

Limitations: No

Major concerns
1. It is important to state a clear hypothesis of this project.

2. In the manuscript we are able to see the number of human subjects included in the study. However, it is unclear how many belong to the BPD group and how many are the controls. The age range is very wide and the cause of demise of the controls is not clear. The number of human subjects with BPD and without needs to be clearly stated as a BPD subject may be paired to multiple controls.

3. Murine hyperoxic models of bronchopulmonary dysplasia are commonly utilized. In a paragraph, it is imperative to note why the authors utilized such a model and the correlation with human disease and lung development.

4. Sex should be considered as a biological variable and data should indicate numbers of males and females

5. Tunicamycin seems to create a lung phenotype resembling the hyperoxic model of bronchopulmonary dysplasia. However, the authors utilize “rat BPD” group as seen in Figure 1. Is “rat BPD” group referring to the hyperoxic model of BPD, Tunicamycin or both? Authors should be careful when interchangeably use BPD in animal designations. Words such as “BPD phenotype” or “Hyperoxic” or any other stressor are preferred so that they do not create confusion.

6. The authors touch upon mitochondrial interactions and report on mitochondrial fission as evidenced by decreased expressions of two major Mitochondrial Associated Membrane proteins and mitochondrial fragmentation. This is mainly displayed in their tunicamycin model and indirectly reported for the hyperoxia model. What were the hyperoxia results of such finding compared to normoxia?

7. Ideally all 4 groups should be displayed in Fig8 (21%O2-PBS, 21%O2-TUDCA,90%O2-PBS, 90%O2- TUDCA)

8. Since the authors state that multiple pharmacological agents may be important to combat BPD it would be novel if they administered both TUDCA and KYC and assess results in their animal Hyperoxic model of BPD. The tunicamycin model will supplement the abovementioned experiment. Groups should be 21%O2-PBS, 21%O2-TUDCA, 21%O2-KYC, 90%O2-PBS, 90%O2- TUDCA, 90%O2- KYC, 90%O2-TUDCA-KYC)

9. Fig4 displays 21%O2-DMSO vs 21%O2-Tunicamycin. This implies experimentation in normoxia as clearly stated in the manuscript. Was Hyperoxia ever used in conjunction to tunicamycin in the experiments?

10.There are numerous limitations of the model. The authors should recognize in a paragraph what these limitations are and why their model of disease is the best to prove their hypothesis.

11. A graphical abstract or graph explaining the experimental design would be greatly helpful.

Minor Concerns

1. Fig 3 needs scale bars; Overlapping graphs and missing “Tunicamycin” Fig 3A.

2. Fig 4A missing “Tunicamycin” ; Fig 4D has a zoomed section; No percentage of zoom is displayed

3. Fig 5A **Bold** font on bar graph

4. Fig6 Overlapping graph titles & inconsistent use of group names (Tunicamycin+PBS vs Tunicamycin-PBS, etc)

5. Fig 8A TUDCA name in bar needs to be corrected.

6. Inconsistent use of abbreviations. Hyperoxia (HOX) is changed in some graphs as “>90%O2” while in others is “HOX” while in others is suspected “ratBPD”.
