# Supplementary material for: Role of endoplasmic reticulum stress in impaired neonatal lung growth and bronchopulmonary dysplasia
Source: PLoS One. 2022 Aug 26;17(8):e0269564. doi: 10.1371/journal.pone.0269564 (PMC9417039; doi:10.1371/journal.pone.0269564)
Supplement: Attachment — Submitted filename: Response to Reviewers.docx [file pone.0269564.s009.docx]

**Dear Dr. Bader:**

Thanks for allowing us to make our manuscript a better one. We sincerely appreciate the reviewers’ valuable comments and have revised our manuscript accordingly. Please see our point-by-point response to the reviewer below.

**Reviewer #1:**

This is a very well-done study with careful attention to rigor and reproducibility. The rationale for exploring the stated links is very strong, and there is substantial new data demonstrating causality in the rat model. The experiments use complementary techniques, and the results are easy to interpret. I have only a few minor concerns.

1. **The authors need to re-examine the figures for neatness. Some of the text are overlapping with images, and there are some font oddities in some of the figures.**

We thank the reviewer for bringing this issue to our attention. The problem we encountered at converting ppt into pdf is resolved. We have gone through all the figures to correct inconsistencies, font, and format problems. We have enclosed a corrected set of figures.

2. **There should be some reference to how the perinatal and neonatal rat lung relates to the human lung developmental stages to show relevance of the hyperoxia rat model and the age groups used to the human condition.**

We agree that the essential information is important for general readers and have added it in the introduction with a relevant reference (lines 70-82).

3. **While the data are clear to someone familiar with the field, for readability, it is critical that an illustrative schematic showing the pathways and links is included as a final figure and/or a graphical abstract.**

Thank you for this great suggestion. We have prepared a figure (Figure 11) to summarize the findings in our manuscript and connected these new data with our previously reported “cycle of destruction”. The figure can be found in the conclusion section (line 570).

**Reviewer #2**

Major concerns

1. **It is important to state a clear hypothesis of this project.**

We have added our hypothesis into the introduction (lines 93-95).

2. **In the manuscript we are able to see the number of human subjects included in the study. However, it is unclear how many belong to the BPD group and how many are the controls. The age range is very wide and the cause of demise of the controls is not clear. The number of human subjects with BPD and without needs to be clearly stated as a BPD subject may be paired to multiple controls.**

We apologize for not clearly stating the 1:1 match, 10 specimens in each group (line 150 and lines 275-276), for the age- and sex-matched human study. We have added a supplemental Table S1 including their age of death, sex, and primary diagnosis.

3. **Murine hyperoxic models of bronchopulmonary dysplasia are commonly utilized. In a paragraph, it is imperative to note why the authors utilized such a model and the correlation with human disease and lung development.**

Thanks for pointing this out. We have added the reason why the rat hyperoxic model was chosen in this study (lines 70-82). As rats are born with a well-developed antioxidant system, we have to use a high concentration of oxygen to elicit lung damage. The pros and cons of using the murine model are added to the revised introduction.

4. **Sex should be considered as a biological variable and data should indicate numbers of**

**males and females.**

Thanks for pointing out the importance of biological variables, especially sex, to the results. We randomly reallocated all pups by sex into 2 or 4 nursing dams to minimize the sex effect in our experiments. The small number of pups in each specific experiment prevented the detection of a statistical difference between the sexes. We thus determined to use data from both sexes for comparisons in each experiment. The number of each sex was added to the results as suggested.

5. **Tunicamycin seems to create a lung phenotype resembling the hyperoxic model of bronchopulmonary dysplasia. However, the authors utilize “rat BPD” group as seen in Figure 1. Is “rat BPD” group referring to the hyperoxic model of BPD, Tunicamycin or both? Authors should be careful when interchangeably use BPD in animal designations. Words such as “BPD phenotype” or “Hyperoxic” or any other stressor are preferred so that they do not create confusion.**

We appreciate the invaluable suggestion to use different descriptors in different situations to avoid confusion for the readers. We have carefully gone over the manuscript to make sure that HOX rat lung and Tun-treated rat lung are used for each condition.

6. **The authors touch upon mitochondrial interactions and report on mitochondrial fission as evidenced by decreased expressions of two major Mitochondrial Associated Membrane proteins and mitochondrial fragmentation. This is mainly displayed in their tunicamycin model and indirectly reported for the hyperoxia model. What were the hyperoxia results of such finding compared to normoxia?**

We previously reported the increased DRP expression in HOX rat lungs as compared to NOX rat lungs (Teng RJ et al. Attenuation of endoplasmic reticulum stress by caffeine ameliorates hyperoxia-induced lung injury. Am J Physiol Lung Cell Mol Physiol. 2017 May 1;312(5):L586-L598.) but did not repeat the same experiment in this study. The information has been added to the results (lines 336-337) and discussion (lines 442-445). We have made the correction in the discussion and referenced that report accordingly.

7. **Ideally all 4 groups should be displayed in Fig8 (21%O2-PBS, 21%O2-TUDCA,90%O2-PBS, 90%O2- TUDCA)**

The 4-group study was performed to compare weight gain, survival rate, and morphometric analyses (**Figure 9**) but we did not do so for the proteomic studies in **Figure 8**. However, the results in Figure 9 support the safety of TUDCA in protecting the rat pups under HOX.

8. **Since the authors state that multiple pharmacological agents may be important to combat BPD it would be novel if they administered both TUDCA and KYC and assess results in their animal Hyperoxic model of BPD. The tunicamycin model will supplement the abovementioned experiment. Groups could be 21%O2-PBS, 21%O2-TUDCA, 21%O2-KYC, 90%O2-PBS, 90%O2- TUDCA, 90%O2- KYC, 90%O2-TUDCA-KYC)**

We deeply appreciate your suggestion and will incorporate into our future studies. Such an experiment with 7 treatment groups will definitely provide important information about treatment strategy and the synergistic/additive effect of two promising chemicals in attenuating BPD. We estimate that such an experiment will require at least 7 dams, and roughly 70 pups, for randomization at the same time which is beyond the capacity of our current animal hyperoxia chamber and space for animals. Our 4-group experiments usually take 2-3 months to accomplish. To pursue the 7-group study, we expect at least 3-5 months will be required. The gestation for SD rats ranges between 21 and 23 days, so the delivery can be 3 days apart between dams which further complicates the randomization. Your suggestion is truly a great idea and will be our future research direction.

9. **Fig4 displays 21%O2-DMSO vs 21%O2-Tunicamycin. This implies experimentation in normoxia as clearly stated in the manuscript. Was Hyperoxia ever used in conjunction to tunicamycin in the experiments?**

Our preliminary experiment showed that rat pups were sensitive to tunicamycin with one injection in the NOX group leading to a relatively high death rate (~40%) by P7. Considering that 20-25% of HOX pups died in our previous experiments, we estimated that a combination of HOX and tunicamycin will lead to a ~50% death rate. We, therefore, did not attempt to use the combined HOX-Tun treatment to study the efficacy of TUDCA. Additionally, we observed the protective effect of TUDCA for the impaired alveolar formation in HOX-alone and Tun-alone treated rat lungs and concluded that these findings provide enough evidence to support the role of ER stress in BPD.

10. **There are numerous limitations of the model. The authors should recognize in a paragraph what these limitations are and why their model of disease is the best to prove their hypothesis.**

We agree that animal models of BPD have limitations. The rat hyperoxia model has been used for decades for studying BPD. The important concerns are the mature antioxidant system in newly born rat pups, unlike the premature infants, and the use of hyperoxia as single stress while BPD in premature infants may have a multi-factorial origin. We addressed these limitations in the section of the revised introduction (lines 70-82).

11. **A graphical abstract or graph explaining the experimental design would be greatly helpful.**

Thank you for the advice. The other reviewer also had a similar suggestion. We created a new figure, figure S1, in our revised manuscript (lines 160-161).

Minor Concerns –

1. **Fig 3 needs scale bars; Overlapping graphs and missing “Tunicamycin” Fig 3A.**

**2. Fig 4A missing “Tunicamycin”; Fig 4D has a zoomed section; No percentage of zoom is displayed**

**3. Fig 5A Bold font on bar graph**

**4. Fig6 Overlapping graph titles & inconsistent use of group names (Tunicamycin+PBS vs Tunicamycin-PBS, etc)**

**5. Fig 8A TUDCA name in bar needs to be corrected.**

We apologize for not realizing that transforming ppt files into pdf files created so many mistakes. We have carefully made corrections for each one of them in the resubmission.

6. **Inconsistent use of abbreviations. Hyperoxia (HOX) is changed in some graphs as “>90%O2” while in others is “HOX” while in others is suspected “rat BPD”.**

Thanks for pointing them out. We have gone over all our figures to make those abbreviations consistent.
